# Prophylaxis after contrast media-induced drug reaction with eosinophilia and systemic symptoms syndrome: our personal experience

**DOI:** 10.1093/ehjcr/ytae514

**Published:** 2024-09-17

**Authors:** Carola Griffith Brookles, Stefano Pizzimenti, Matteo Bianco

**Affiliations:** Cardiology Division, San Luigi Gonzaga University Hospital, Regione Gonzole 10, Orbassano, Turin, 10043, Italy; Department of Medical Sciences, University of Turin, Turin, Italy; Severe Asthma and Rare Lung Disease Unit, San Luigi Gonzaga University Hospital, Turin, Italy; Cardiology Division, San Luigi Gonzaga University Hospital, Regione Gonzole 10, Orbassano, Turin, 10043, Italy

As highlighted in the letter we received from Dr Boehm,^1^ to which we are willing to respond, premedication with corticosteroids is a controversial yet diffused strategy to prevent re-occurrences of iodinated contrast medium (ICM)-related allergic reactions (*Figure 1*). In a landmark randomized clinical trial (RCT),^2^ premedication with a standardized regimen of methylprednisolone significantly reduced allergic reactions to high-osmolality contrast media. However, these results were not confirmed in subsequent trials implying the use of low-osmolality ICMs.

**Figure 1:**
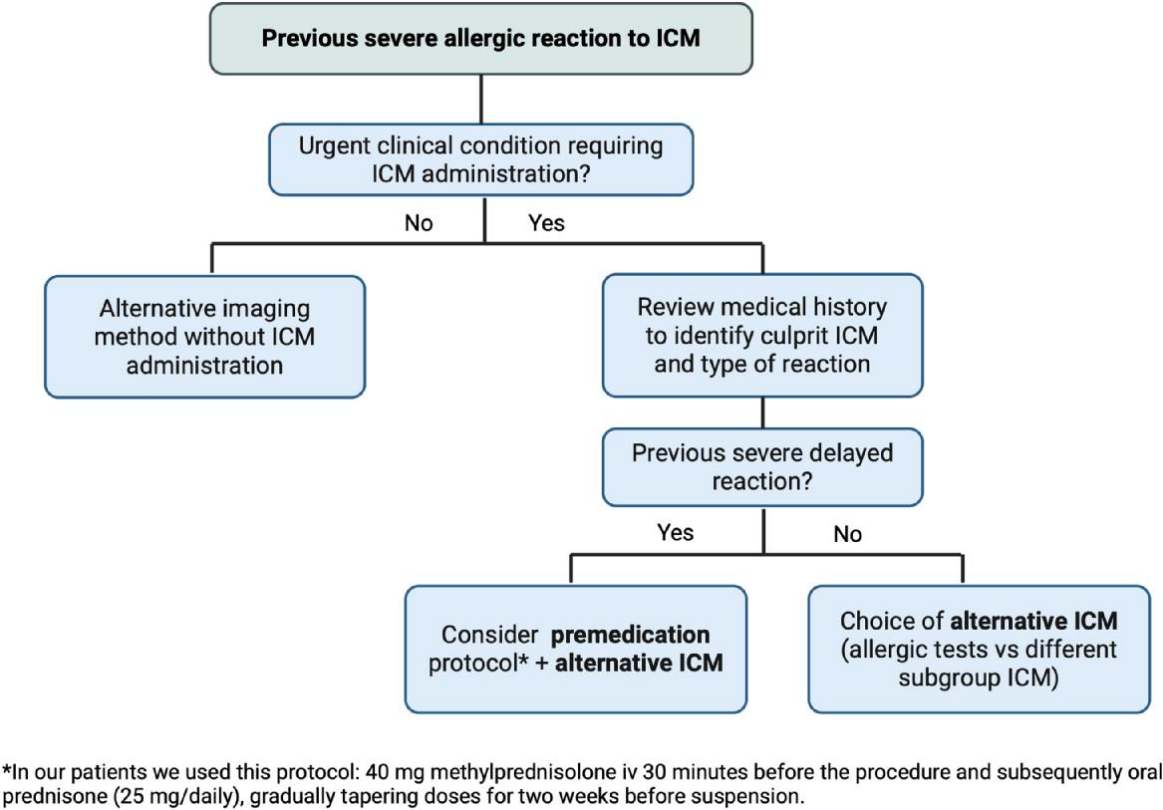
Proposed strategy for patients with history of severe allergic reactions to iodinated contrast medium requiring re-administration. We suggest that premedication should be considered for patients who experienced severe delayed allergic reactions to iodinated contrast medium, together with the choice of an alternative iodinated contrast medium when the culprit is known. ICM, iodinated contrast medium; IV, intravenously; vs, versus.

Corticosteroids, albeit extensively available, may provide a false sense of security without fully protecting towards breakthrough reactions while exposing to side effects. Studies including patients with previous immediate reactions to ICM did not show a significant benefit from premedication when patients were re-exposed to the index ICM, while switching ICM was effective in reducing re-occurrences. However, sample numbers were limited, premedication regimens lacked standardization, and the absence of randomization could have mitigated the benefits of this strategy.^3^

Evidence regarding premedication mainly refers to immediate allergic reactions to ICM, while patients we presented had a history of drug reaction with eosinophilia and systemic symptoms (DRESS) syndrome, a severe non-immediate allergic reaction. In this setting, patch testing, intradermal testing, and lymphocyte activation assays may be used to confirm drug causality, but test positivity is highly dependent on the type of drug and test protocol, with variable predictive values.^4^

Premedication protocols need standardization; in the cases we presented, both patients received 40 mg methylprednisolone intravenously 30 min before the procedure and subsequently switched to oral corticosteroid (prednisone 25 mg/daily), gradually tapering doses for 2 weeks before suspension.

It has been now validated that allergic reactions are not elicitated by iodine or iodinicity, rather by the structure of carbamoyl side chains, and it is not uncommon for patients to develop a positive reaction to more than one ICM. The term ‘polyvalent reactivity’ generically refers to the presence of multiple positive results in skin tests. When positive results involve ICMs sharing common molecular structures, the expression ‘cross-reactivity’ can be used. However, this term is frequently used to describe the presence of multiple positive results, independently from molecular similarities. In one of our cases, the choice of an alternative ICM was led by Lerondeau *et al.*^5^ classification, based on chemical structure.

Some studies have highlighted that, when allergic patients are tested to different ICMs, ‘cross-reactivity’ is rarer than expected, prompting questions about the existence of an individual reaction pattern (either a mono- or polyvalent) and which classification is more accurate to select an alternative ICM when allergic testing results are unavailable.^6^ More studies are needed to clarify all these aspects.

In our personal experience, the choice of an alternative ICM (based on results of allergic testing when available or by Lerondeau’s classification), together with the use of a standardized corticosteroid premedication protocol, was effective in preventing re-occurrences of DRESS syndrome, without significant side effects. We recognize the need for RCTs to definitively assess the role of premedication in high-risk patients exposed to low-osmolar ICM, in addition to the change of the culprit ICM. An effort to enhance the diffusion of allergic testing, together with a better identification and registration of the culprit ICM in medical records is essential. The definition of the optimal strategy to prevent re-occurrences is necessary to guarantee potentially life-saving procedures while preserving safety of patients.

## Data Availability

The data underlying this article are available in the article. Additional data could be provided upon reasonable requests.

## Notes

**Funding:** None declared.
